# 2847. Healthcare utilization among older adults hospitalized with complicated urinary tract infections in the United States

**DOI:** 10.1093/ofid/ofad500.2457

**Published:** 2023-11-27

**Authors:** Helen L Zhang, Tetsu Ohnuma, Alexandria A Spellman, Karthik Raghunathan, Vijay Krishnamoorthy, Deverick J Anderson, Kenneth E Schmader, Charles Scales, Sonali Advani

**Affiliations:** Duke University, Durham, North Carolina; Duke University, Durham, North Carolina; Duke University Medical Center, Durham, North Carolina; Duke University, Durham, North Carolina; Duke University, Durham, North Carolina; Duke Center for Antimicrobial Stewardship and Infection Prevention, Durham, North Carolina; Duke and Durham VA Medical Centers, Durham, North Carolina; Duke University School of Medicine, Durham, North Carolina; Duke University School of Medicine, Durham, North Carolina

## Abstract

**Background:**

Healthcare utilization for complicated urinary tract infections (cUTI) is increasing in recent years, especially among older adults due to comorbidities and immunosuppression. However, healthcare utilization and inpatient antibiotic use for cUTI are poorly described among older adults.

**Methods:**

This retrospective cohort study included inpatients aged ≥18 years with cUTI identified using ICD-10 diagnostic codes who were discharged alive from 240 hospitals in the Premier Healthcare Database (January 2016 – June 2020). Sociodemographic factors, clinical characteristics, antibiotic use, and healthcare utilization measures were stratified into 2 age groups: adults 65 years and older (“older adults”), and adults under 65 years (“younger adults”). Bivariate comparisons were performed using chi-square test for categorical variables and Wilcoxon rank sum test for continuous variables.

**Results:**

Among 14,981 patients, 8,073 (53.9%) were ≥65 years. Compared to younger adults, older adults with cUTI were more likely to be male (54.5% vs. 38.1%, p < 0.001), be in an intensive care unit (70.4% vs. 65.7%, p < 0.001), and receive vasopressors (26.6% vs. 23.1%, p < 0.001). Older adults with cUTI had a longer median length of stay (8 vs. 7 days, p < 0.001), were more likely to be discharged to nursing homes (40.7% vs. 18.2%, p < 0.001), and had a higher median cost of hospitalization ($18,191.62 vs. $15,800.76, p < 0.001). Older adults also received higher median days of antibiotic therapy (11 vs. 10 days, p = 0.009) and were more likely to receive intravenous (IV) vancomycin (54.9% vs. 51.7%, p < 0.001) or cefepime (31.2% vs. 28.0%, p < 0.001) though less likely to receive an aminoglycoside (9.5% vs. 12.1%, p < 0.001) (Table 1).

**Figure:**
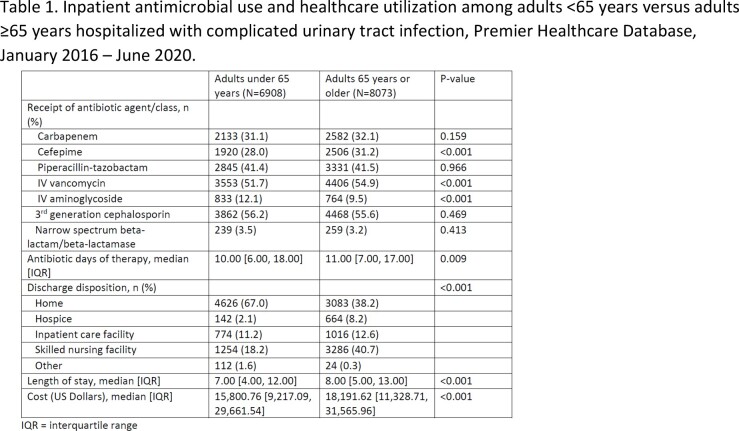

**Conclusion:**

Older adults hospitalized with cUTI required higher acuity of care and more post-acute institutional care, and they incurred higher healthcare costs than younger adults. Older adults also had different antimicrobial utilization patterns, including more frequent use of IV vancomycin or cefepime. Future studies should evaluate post-discharge healthcare utilization and antimicrobial use among older adults with cUTI.

**Disclosures:**

**Charles Scales, MD MSHS**, BMS: Grant/Research Support|Exelixis: Grant/Research Support|Flume Catheter: Grant/Research Support|Merck: Grant/Research Support|Pfizer: Grant/Research Support **Sonali Advani, MBBS, MPH, FIDSA**, bioMérieux: Advisor/Consultant|GlaxoSmithKline: Advisor/Consultant

